# Elevated serum gamma-glutamyltransferase is associated with an increased risk of oesophageal carcinoma in a cohort of 8,388,256 Korean subjects

**DOI:** 10.1371/journal.pone.0177053

**Published:** 2017-05-05

**Authors:** Yoon Jin Choi, Dong Ho Lee, Kyung-Do Han, Hyuk Yoon, Cheol Min Shin, Young Soo Park, Nayoung Kim

**Affiliations:** 1 Department of Internal Medicine and Seoul National University Bundang Hospital, Seongnam, Gyeonggi-do, South Korea; 2 Department of Internal Medicine and Liver Research Institute, Seoul National University College of Medicine, Seoul, South Korea; 3 Department of Biostatistics, College of Medicine, The Catholic University of Korea, Seoul, South Korea; National Health Research Institutes, TAIWAN

## Abstract

Gamma-glutamyltransferase (GGT) is a marker for hepatic injury and alcohol consumption. However, the association of GGT with the risk of oesophageal carcinoma (OC) has not been fully recognized to date. Therefore, this study aimed to determine the association between elevated GGT and OC, by also considering the body mass index (BMI) of the subjects. Clinical data from 8,388,256 Korean individuals, who were aged 40 years and over and who received healthcare check-ups arranged by the national insurance program in 2007 and 2008, were analysed. Newly diagnosed OC was identified using claims data during a median follow-up duration of 8.72 years. During the study period, 6,863 individuals (0.08%) developed OC. We found that there was an increased risk of OC in subjects with serum GGT values >18 IU/L. Furthermore, a BMI <18.5 kg/m^2^ (underweight) was associated with increased OC risk, while a BMI ≥23.0 kg/m^2^ was associated with a reduced OC risk. Individuals who were both underweight and in the highest GGT quartile (≥40 IU/L) had a far greater risk of OC compared to other individuals (hazard ratio: 3.65, 95% confidence interval: 3.10–4.30). In conclusion, increased serum GGT was associated with an increased risk of developing OC in the general Korean population, regardless of age, sex, smoker status, or alcohol consumption.

## Introduction

Oesophageal cancer (OC) is the tenth most common cancer and the sixth leading cause of cancer-related death worldwide [1]. OC usually has a poor prognosis, because most lesions are detected at an advanced stage; the tumours can quickly invade and spread into the oesophagus and adjacent organs. Although upper gastroendoscopy is commonly performed, it is difficult to detect early-stage lesions. Moreover, no specific markers for the early detection of OC have yet been discovered.

There are geographical differences in the prevalence of different pathological types of OC. The two most common histopathological types are oesophageal adenocarcinoma (OAC) and oesophageal squamous cell carcinoma (OSCC). OAC is more common in Western countries [1], whereas OSCC is more prevalent in East Asia, including Korea [2]. In fact, OSCC accounts for 95% of OC cases in Korea [2]. While OAC is considered an obesity-related disease, as obesity is one of its risk factors along with gastroesophageal reflux and Barrett’s oesophagus [3], OSCC is considered to have distinct risk factors, including smoking, excessive alcohol consumption, and hot tea consumption [3,4]. However, these risk factors are not yet fully understood.

Gamma-glutamyltransferase (GGT) is a marker of liver injury; however, it is less sensitive compared with alanine aminotransferase (ALT) or aspartate aminotransferase (AST), and is therefore seldom used for detecting or monitoring liver diseases. Recently, however, the roles of GGT as a prognostic indicator for other diseases such as type 2 diabetes mellitus (DM) [5,6], metabolic syndrome [7], and cardiovascular disease [5], as well as for all-cause mortality [8], have been reported. Moreover, high serum values of GGT, including the upper limits of the normal range, are associated with increased risks of cancers originating from various organs, including the breast [9], prostate [10], digestive system [11], and respiratory system [12].

With this in mind, the present study aimed to investigate the association between the GGT level and the incidence of OC in Korea using population-based national data. Furthermore, to date, only a limited number of studies have evaluated the relationship between being underweight and OSCC risk [13,14], which comprises 95% of OCs in Korea [2]. Thus, the secondary objective was to identify whether underweight status is associated with OC in Korea and whether a low body mass index (BMI) additionally increases the risk of OC in individuals with high GGT.

## Materials and methods

### Data source and study population

We used the database of the National Health Insurance Corporation (NHIC), which is a national insurer managed by the Korean government and to which approximately 97% of the Korean population subscribes [15]. The NHIC subscribers are recommended to undergo a standardized medical examination biennially. Any researcher can use the NHIC data if the study protocols are approved by the official review committee.

**Fig 1:**
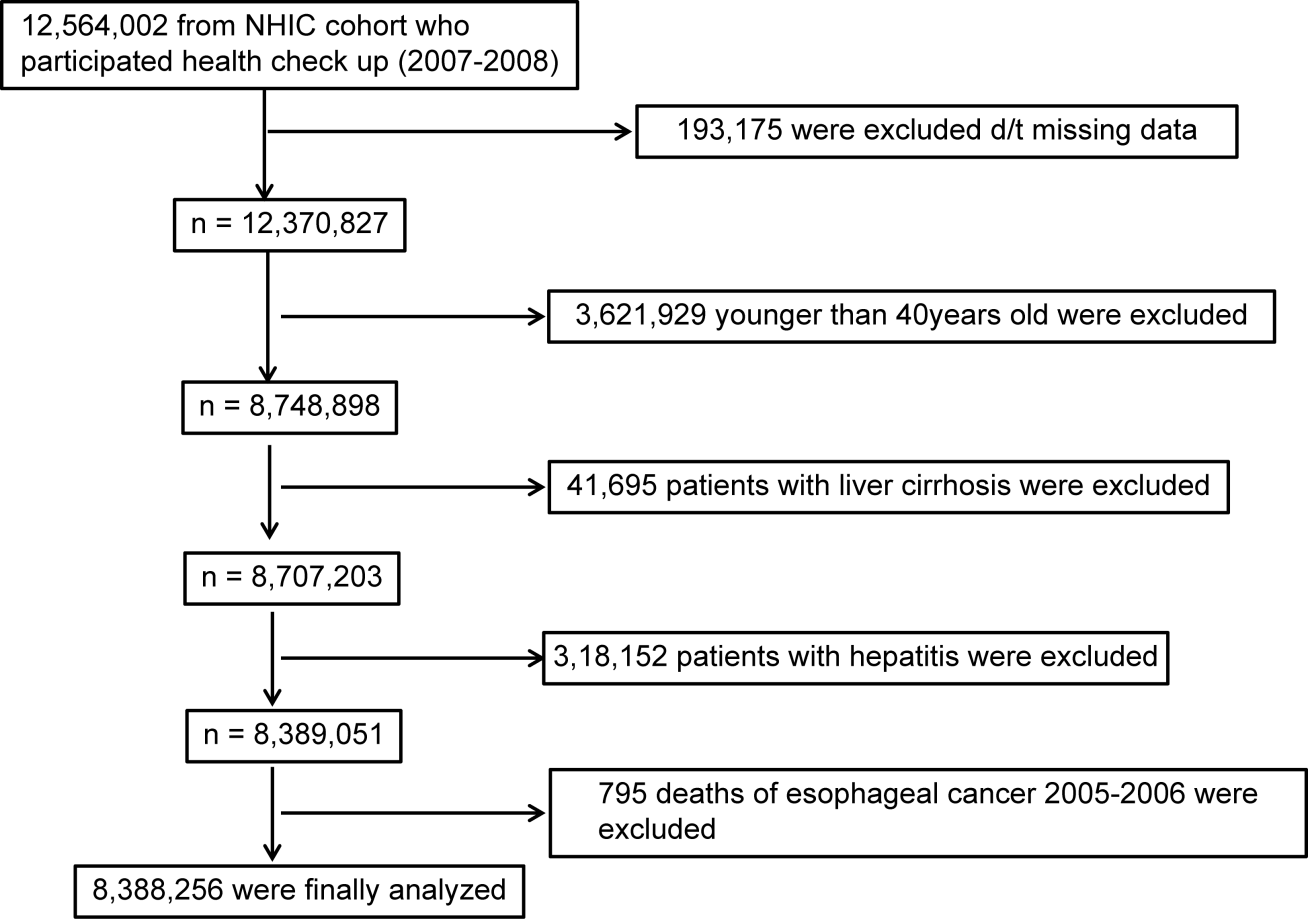
Flowchart showing the enrolment process for the study cohort. NHIC, National Health Insurance Corporation.

Among 12,564,002 individuals who had undergone a biennial evaluation provided by the NHIC in 2007 and 2008, data from the medical records of 8,748,898 individuals aged ≥40 years were evaluated. The primary endpoint of this study was newly diagnosed OC, which was defined using the International Classification of Diseases, 10th revision (ICD-10) codes (C150-155, C158, and C159). Enrolment in both systems was required for diagnosis. To avoid enrolling patients with pre-existing disease, individuals diagnosed with OC during 2005 and 2006 were excluded. All patients with a previous malignancy were also excluded. Patients with liver cirrhosis (K703) and hepatitis (K746) were excluded to avoid the potential confusion caused by liver disease-related factors, and the data were further stratified into drinker and non-drinker groups. A summary of the study population selection is illustrated in Fig 1.

### Data collection

Standardized self-reporting questionnaires were used to collect data at baseline for the following variables, which are regarded as risk factors for liver injury and were included as covariates in the multivariable analyses: age (years), sex, residency (rural and urban), yearly income (lowest quintile vs. the remaining quintiles), alcohol intake (frequency: never or near abstinence, 2–3 times/month, 1–2 times/week, 3–4 times/week, and ≥5 times/week; and amount: complete or near abstinence, <3, 6, 9, or 12 standard drinks of 10 g alcohol units per drink), cigarette smoking (never, former, and current), and physical activity level (low, moderate, or high). BMI and systolic and diastolic blood pressure (mmHg) were also measured. Subjects were categorized into the following four BMI groups according to the World Health Organization recommendations for the Asian population: underweight, <18.5 kg/m^2^; normal, 18.5–22.9 kg/m^2^; overweight, 23.0–24.9 kg/m^2^; and obese, ≥25.0 kg/m^2^ [16].

Values of total cholesterol (mg/dL) and liver enzymes, such as ALT, AST, and GGT, in the serum (IU/L) were determined. GGT was measured mainly by the Szasz method under Korean Association of Laboratory Quality Control Standards [17].

### Statistical analyses

GGT values were stratified into four quartiles: ≤16, 17–23, 24–39, and ≥40 IU/L. Data are presented as the mean ± SD for normally distributed continuous variables and as proportions for categorical variables. The Student t-test and ANOVA were used to analyse continuous variables, and the differences between nominal variables were compared with the chi-square test. The incidence rates of OC were calculated by dividing the number of events by the person-time at risk. To determine the independent association of GGT with the risk of OC incidence, the Cox proportional hazards model was used after adjusting for age, smoking status, alcohol consumption, exercise, and BMI. Subgroup analyses were performed according to age, sex, smoking status, drinking habits, and presence or absence of diabetes.

All statistical analyses were performed using SAS version 9.4 (SAS Institute, Cary, NC, USA) and R version 3.2.3 (The R Foundation for Statistical Computing, Vienna, Austria, http://www.Rproject.org). A two-sided p-value of less than 0.05 was considered statistically significant.

### Ethical approval

Because this study involved routinely collected data, informed consent was not specifically obtained. The study was approved by the Institutional Review Board of Seoul National University Bundang Hospital (X-1608/360-904). All procedures involving human participants were performed in accordance with the ethical standards of the institutional and national research committees and with the 1964 Helsinki declaration, including its later amendments, or comparable ethical standards.

## Results

### Demographic characteristics

Of the 8,388,256 subjects included in the analysis, 6,863 (0.08%) developed OC. The median follow-up period was 8.72 years, and the median time to OC development was 4.00 years. The geometric mean GGT value was 26.7 IU/L and the median (interquartile range) was 24.0 (16.0–39.0) IU/L; for the analysis, the subjects were divided into four quartiles based on their serum GGT values (Table 1).

**Table 1:** Demographics of the study enrolees.

Serum GGT value^a^	Q1(≤16 IU/L)	Q2 (17–23 IU/L)	Q3 (24–39 IU/L)	Q4 (≥40 IU/L)	*p*
	(n = 2251868)	(n = 1905085)	(n = 2145903)	(n = 2085400)	
Age (y, mean±SD)	54.9±10.7	56.1±10.6	55.6±10.2	53.8±9.4	< .0001
(y, range) 40–49	890951(39.56)	622381(32.67)	707656(32.98)	812724(38.97)	< .0001
50–64	940567(41.77)	881805(46.29)	1032607(48.12)	992522(47.59)	
65-	420350(18.67)	400899(21.04)	405640(18.9)	280154(13.43)	
Sex (Male), n (%)	401962(17.85)	755041(39.63)	1319221(61.48)	1692574(81.16)	< .0001
Current/ex-smoker, n (%)	138336(6.14)	258908(13.59)	487952(22.74)	756474(36.27)	< .0001
Alcohol consumption, n (%)	478562(21.25)	588977(30.92)	956979(44.6)	1381461(66.24)	< .0001
Exercise, n (%)	235692(10.47)	207575(10.9)	225210(10.49)	186024(8.92)	< .0001
Lower quintile of yearly income, n (%)	512592(22.76)	398528(20.92)	417350(19.45)	390604(18.73)	< .0001
Residence area (rural)	1192219(52.94)	1019851(53.53)	1148508(53.52)	1129299(54.15)	< .0001
Diabetes, n (%)	130972(5.82)	174865(9.18)	266660(12.43)	351909(16.87)	< .0001
Hypertension, n (%)	572077(25.40)	630317(33.09)	813762(37.92)	912370(43.75)	< .0001
Dyslipidaemia, n (%)	303573(13.48)	359775(18.88)	472053(22.00)	535824(25.69)	< .0001
Developing *oesophageal cancer*, n (%)	717(0.03)	1058(0.06)	1775(0.08)	3313(0.16)	< .0001
BMI, kg/m2	23±2.8	23.7±2.9	24.3±2.9	24.9±3.0	< .0001
BMI < 18.5 Kg/m2, n (%)	75508(3.35)	48921(2.57)	38085(1.77)	28289(1.36)	< .0001
SBP, mmHg (mean±SD)	121.5±16.1	124.4±16.1	126.3±15.8	129.1±15.9	< .0001
DBP, mmHg (mean±SD)	75.1±10.2	77±10.2	78.5±10.1	80.8±10.4	< .0001
Sr glucose, mg/dL (mean±SD)	93.4±18.2	96.8±22.3	100.3±26.2	105.7±30.9	< .0001
Sr cholesterol, mg/dL (mean±SD)	191.5±34.5	197.5±36.0	200.5±36.6	204±38.7	< .0001
AST, (IU/L)^b^	20.72 (20.71–20.73)	22.06 (22.05–22.07)	23.81 (23.81–23.82)	29.67 (29.66–29.69)	< .0001
ALT, (IU/L)^b^	15.83 (15.82–15.84)	18.76 (18.75–18.77)	22.63 (22.62–22.64)	31.48 (31.45–31.50)	< .0001
GGT, (IU/L)^b^	12.46 (12.46–12.46)	19.67 (19.66–19.67)	29.81 (29.80–29.81)	71.47 (71.42–71.53)	< .0001

GGT, gamma-glutamyltransferase; BMI, body mass index; SBP, systolic blood pressure; DBP, diastolic blood pressure; AST, aspartate aminotransferase; ALT, alanine transaminase; SD, standard deviation.

^a^Q1-4 denotes the quartile of the serum value of gamma-glutamyltransferase.

^b^ Geometric mean (95% confidence interval).

Compared to the lower-quartile groups (Q1), the higher-quartile GGT groups (Q1 vs. and Q2-4) included more men, as well as subjects who smoked and consumed alcohol more frequently, had higher income levels, and were residents of urban areas. Moreover, subjects in the higher-quartile GGT groups had higher blood pressure, fasting glucose, total cholesterol, AST, ALT, and BMI values compared with those in the lower-quartile GGT groups.

#### Multivariable analyses for risk factors associated with the development of OC

**Fig 2:**
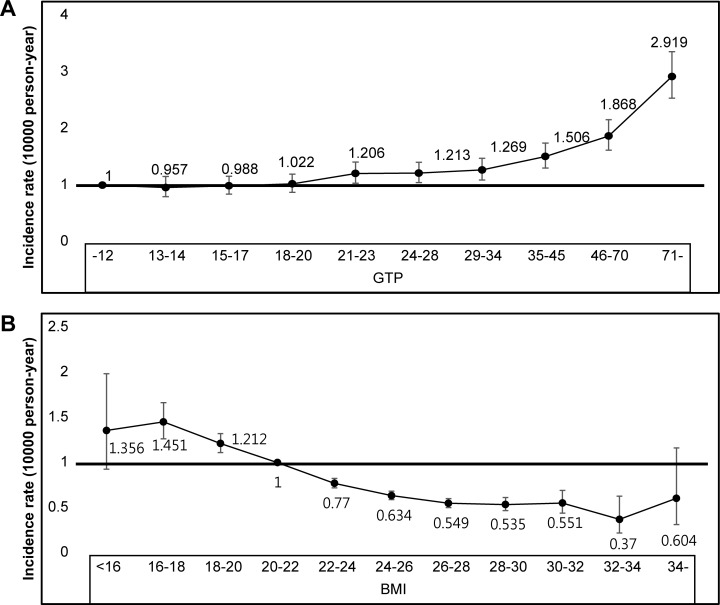
Association between the Hazard Ratio for Oesophageal Cancer and the Serum Gamma-glutamyltransferase Level (A) and Body Mass Index (B) in the General Korean Population.

The risks of developing OC according to the GGT quartiles and graded BMIs are shown in Fig 2; the risk of OC development continuously increased with rising GGT values. Moreover, subjects with a BMI <22.0 kg/m^2^ showed an increased risk of developing OC, whereas those with a BMI >22.0 kg/m^2^ were less likely to develop OC.

**Fig 3:**
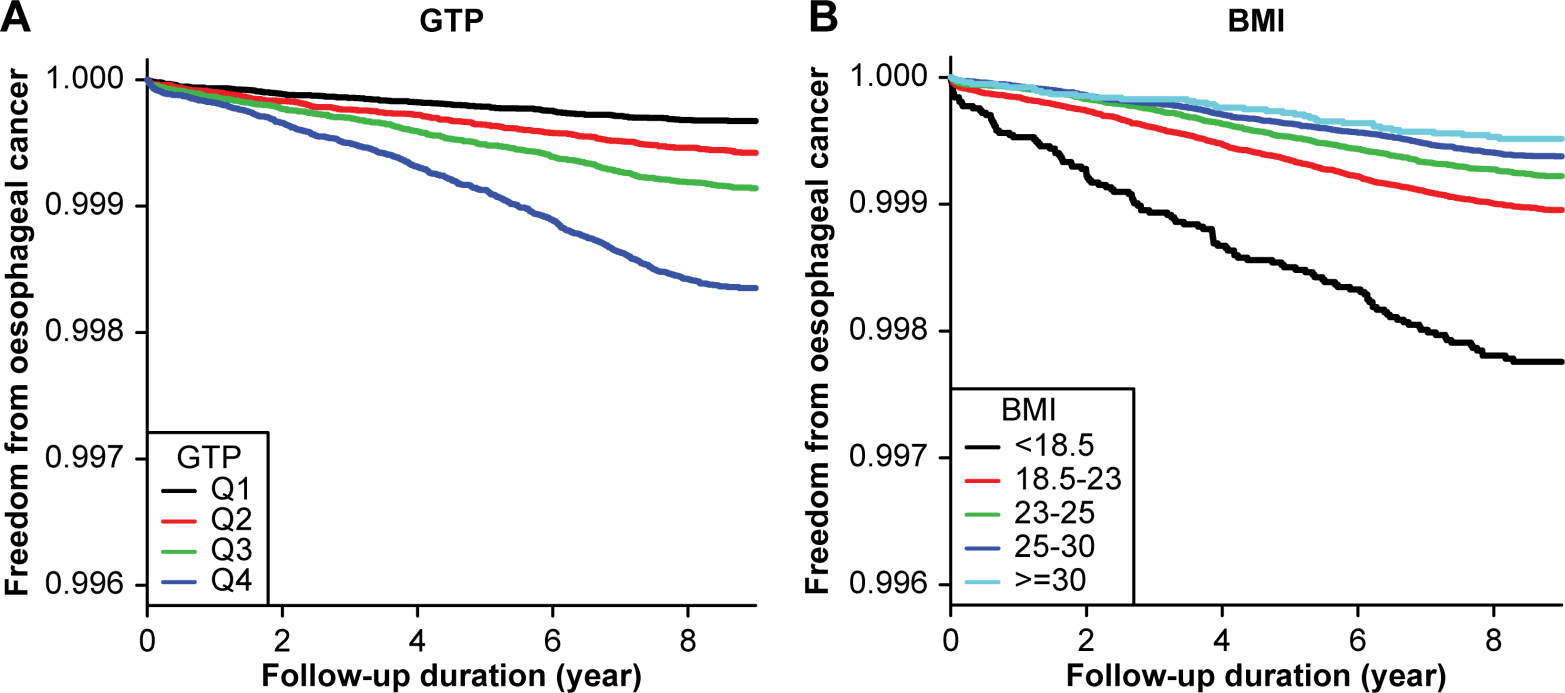
Kaplan-Meier survival curves showing freedom from oesophageal cancer in Korea. Q1-4 denotes the quartile of the serum value of gamma-glutamyltransferase (GGT). The incidence is presented as the annual incidence rate per 10,000 person-years, adjusted for age, sex, body mass index (BMI), smoking, drinking, exercise, income, residence locale, diabetes, hypertension, and dyslipidaemia. Subjects with (A) the highest serum GGT quartile and (B) lowest BMI quartile (underweight) showed the highest incidences of oesophageal cancer.

Table 2 shows the results of two multivariable models used to evaluate the risk factors of OC. In the first model, adjusted for age and sex, serum GGT >17 IU/L and BMI <18.5 kg/m^2^ were significantly associated with increased risks of developing OC. BMI ≥23.0 kg/m^2^ was associated with a decreased risk of OC. In the second multivariable model, adjusted for age, sex, BMI, smoking, alcohol consumption, exercise, income, residency locale, DM, hypertension, and dyslipidaemia, similar results were obtained. That is, the highest incidences of OC were seen in individuals with high serum GGT values and low BMI (Fig 3). BMI ≥23.0 kg/m^2^ was associated with a reduced OC risk compared to the normal range of BMI. After excluding the first 2 years of follow-up, upregulated serum GGT or low BMI remained associated with an increased risk of developing OC (S1 Table).

**Table 2:** Multivariable analyses of the impact of the serum GGT level and BMI on the risk of oesophageal cancer in the general Korean population.

Variable				HR (95% CI)	
	Event	Duration	IR^a^	Model 1^b^	Model 2^c^
**GGT**^**d**^					
Q1	717	19734967.07	0.3633	1.00 (ref.)	1.00 (ref.)
Q2	1058	16638104.39	0.6359	1.07 (0.97–1.17)	1.09 (0.99–1.20)
Q3	1775	18703651.66	0.9490	1.24 (1.14–1.36)	1.29 (1.18–1.42)
Q4	3313	18065974.75	1.8338	2.26 (2.07–2.45)	2.22 (2.03–2.43)
**BMI**					
<18.5	384	1566026.18	2.4521	1.42 (1.28–1.58)	1.42 (1.28–1.58)
18.5–23	3116	26376761.36	1.1813	1.00 (ref.)	1.00 (ref.)
23–25	1682	20060005.8	0.8385	0.70 (0.66–0.74)	0.71 (0.66–0.75)
25–30	1577	22934861.75	0.6876	0.59 (0.55–0.62)	0.59 (0.55–0.62)
30-	104	2205042.76	0.4716	0.56 (0.46–0.68)	0.54 (0.45–0.66)

GGT, gamma-glutamyltransferase; BMI, body mass index; HR, hazard ratio; CI, confidential interval.

^a^ Per 10,000 person-year

^b^Model 1: Adjusted for age and sex

^c^Model 2: Adjusted for age, sex, BMI, smoking, drinking, exercise, income, residence locale, diabetes, hypertension, and dyslipidaemia.

^d^GGT values were stratified into 4 quartiles: ≤16, 17–23, 24–39, and ≥40 IU/L.

#### Effect of high GGT values combined with underweight status on the development of OC

Comparison of the four combinations of the presence/absence of the highest GGT quartile (≥40 IU/L) and presence/absence of underweight status (BMI <18.5 kg/m^2^) showed that the co-existence of the two factors was associated with a significantly greater risk of OC compared with the other combinations (hazard ratio: 3.65, 95% confidence interval: 3.10–4.30) after adjusting for age, sex, BMI, smoking, alcohol consumption, exercise, income, residency, DM, hypertension, and dyslipidaemia. The incidence rate of OC in subjects who were in the highest GGT quartile and who were also underweight was 6.97 per 10,000 person-years, whereas the rate among subjects who did not fit into either category was <2 per 10,000 person-years.

#### Stratified analyses according to age, sex, smoking status, and alcohol consumption

Table 3 shows the incidence rates and hazard ratios of OC according to the subjects’ age range, sex, smoking status, drinking status, and DM. In subjects aged ≥55 years, GGT ≥40 IU/L, underweight status, and the simultaneous presence of both factors were associated with increased risks of developing OC. When the subjects’ ages were further divided into 5-year increments, being simultaneously underweight and within the highest GGT quartile was associated with a markedly elevated risk of developing OC across all age ranges (S2 Table). Subjects in the age range of 40–49 years showed the highest risk of developing OC compared to the older age groups (hazard ratio: 11.68, 95% confidence interval: 6.51–20.96).

**Table 3:** Subgroup analyses of the impact of the GGT level and underweight status on the risk of oesophageal cancer in the general Korean population.

Variables			Q4^a^ (-) UW(-)	Q4^a^ (+) UW(-)	Q4^a^ (-) UW(+)	Q4^a^ (+) UW(+)	P for interaction
**Total**		IR	0.62	1.77	1.71	6.97	
		HR^b^ (95% CI)	1 (ref.)	1.74 (1.65–1.84)	1.64 (1.44–1.88)	3.65 (3.10–4.30)	
**Age**	Age <55years	IR	0.18	0.66	0.52	3.19	< .001
		HR^b^ (95% CI)	1 (ref.)	1.84 (1.63–2.08)	3.26 (2.31–4.60)	7.72 (5.38–11.08)	
	Age ≥55years	IR	1.17	3.56	2.92	10.13	
		HR^b^ (95% CI)	1 (ref.)	1.70 (1.60–1.80)	1.63 (1.41–1.89)	3.22 (2.68–3.87)	
**Sex**	Men	IR	1.36	2.13	3.56	8.53	0.127
		HR^b^ (95% CI)	1 (ref.)	1.77 (1.68–1.87)	1.55 (1.33–1.79)	3.65 (3.09–4.30)	
	Women	IR	0.14	0.23	0.46	0.68	
		HR^b^ (95% CI)	1 (ref.)	1.41 (1.11–1.80)	2.51 (1.79–3.53)	3.02 (0.97–9.40)	
**Smoking**	No	IR	0.47	1.48	1.31	4.95	0.016
		HR^b^ (95% CI)	1 (ref.)	1.84 (1.72–1.97)	1.86 (1.56–2.21)	3.55 (2.68–4.69)	
	Current /ex	IR	1.52	2.29	3.21	8.74	
		HR^b^ (95% CI)	1 (ref.)	1.58 (1.45–1.71)	1.36 (1.10–1.69)	3.45 (2.81–4.23)	
**Drinking**	No	IR	0.47	1.05	1.29	4.52	0.363
		HR^b^ (95% CI)	1 (ref.)	1.52 (1.40–1.65)	1.67 (1.40–1.98)	3.71 (2.74–5.02)	
	Yes	IR	1.25	2.37	3.88	8.83	
		HR^b^ (95% CI)	1 (ref.)	1.74 (1.62–1.87)	1.66 (1.33–2.07)	3.34 (2.74–4.06)	
**Diabetes**	No	IR	0.58	1.70	1.63	6.58	0.384
		HR^b^ (95% CI)	1 (ref.)	1.77 (1.67–1.88)	1.60 (1.39–1.85)	3.51 (2.93–4.19)	
	Yes	IR	1.04	2.12	3.17	9.89	
		HR^b^ (95% CI)	1 (ref.)	1.60 (1.41–1.81)	2.02 (1.33–3.08)	4.46 (2.99–6.65)	

UW, underweight (body mass index < 18.5 kg/m^2^); IR, incidence rate; HR, hazard ratio; CI, confidential interval; ref, reference.

^a^Q4, the highest quartile of serum gamma-glutamyltransferase (≥40 IU/L)

^b^Adjusted for age, sex, body mass index, smoking, drinking, exercise, income, residence locale, diabetes, hypertension, and dyslipidaemia.

Finally, our data showed that a serum GGT level ≥40 IU/L, a BMI <18.5 kg/m^2^, and simultaneous presence of both factors were uniform risk factors associated with developing OC even after adjusting for sex, smoking status, alcohol consumption, and DM (Table 3). These effects were modified by the subjects’ age and smoking status (p for interaction <0.001 and 0.016, respectively).

## Discussion

In the present study of the general Korean population, we found that the risk of OC steadily increased as the serum GGT level rose above 18 IU/L, even after controlling for confounding factors such as alcohol consumption and smoking. Underweight status was also a risk factor for OC in Korea. Moreover, there was a synergistic increase in OC risk when the serum GGT level was >40 IU/L and BMI was <18 kg/m^2^.

Our observation of an increased susceptibility to OC in subjects with an elevated GGT level is consistent with the results of previous studies [13,14]. However, the underlying mechanisms that explain this phenomenon have not been fully elucidated. Since GGT elevation is strongly associated with heavy alcohol use [18], the increased risk of OC could potentially be explained by heavy drinking. A previous study showed that there was a positive relationship between GGT and alcohol-related cancers among current drinkers [19]. The carcinogenic potential of alcoholic beverages to cause OC may be due to ethanol itself, as well as acetaldehyde, which is a carcinogen derived from ethanol metabolism [20]. In the present study, however, GGT was associated with an elevated risk of OC regardless of the level of alcohol consumption.

Another well-supported hypothesis for elevated serum GGT values is that this enzyme plays a role in the cellular response to oxidative stress [21,22]. GGT plays a pivotal role in the extracellular catabolism of glutathione, a powerful antioxidant, and is upregulated in response to oxidative stress in epithelial and cancer cells. Accordingly, the transcription of GGT is increased after exposure to oxidants [23,24]. It has been suggested that elevation of GGT is an adaptive response of cancer cells for protection against oxidative stress [25].

It has also been shown that GGT is dysregulated in malignant cells. The production of reactive oxygen species, facilitated by GGT through a separate mechanism, leads to tumour progression towards a more aggressive phenotype that is associated with a worse prognosis [26,27]. High GGT activity was shown to be associated with substantial levels of background DNA damage in human GGT-transfected melanoma cells [28]. Moreover, it has recently been reported that a high serum GGT value is independently associated with an increased burden of subclinical inflammation [29]. Taken together, all of these events may promote tumourigenesis.

Several epidemiologic studies support the association between underweight status and OSCC [30,31]. Poor diet and malnutrition, commonly observed in individuals with low BMI, can lead to micronutrient deficiencies and have been implicated as high-risk factors for OSCC, specifically in Asian populations [14,32]. Although several studies have revealed an inverse relationship between BMI and OSCC risk, they often fail to demonstrate the effect of underweight status on OSCC with respect to smoking. However, our large national cohort study clearly demonstrated that being underweight is associated with an increased incidence of OC (predominantly OSCC in Korea) in both sexes and in both non-drinkers and non-smokers (S3 Table). The underlying reason for the synergistic effect of increased GGT and underweight status on the susceptibility to OC might be oxidative stress amplified by undernutrition [33,34]. Under- or malnutrition is usually associated with a lack of nutrients required for antioxidant or immune functions. However, in this study, interactions of age and smoking with the effect of the highest quartile of GGT and underweight status on developing OC were observed. That is, for individuals aged <55 years, a markedly increased risk of OC was seen in subjects with both the highest quartile of GGT and underweight status, as compared to in those aged over 55 years. This may be mainly because healthy young adults are less likely to be underweight than elderly (S3 Table). On the other hand, in non-smokers, consistently higher risks of OC were seen among all combinations of the two factors. Because cigarette smoking is known to be significantly associated with increased levels of GGT [35,36], the unexpected elevation of GGT in non-smokers may reflect a stronger association between the GGT level and OC compared to in smokers.

Furthermore, although a lower risk of death for both OSCC and OAC has been reported in patients with a pre-diagnosis BMI of ≥ 25 kg/m^2^ [37], studies about the protective effects of high BMI on developing OSCC are limited. In the present study, a pre-diagnosis BMI ≥23.0 kg/m^2^ was associated with a reduced OC risk compared to the normal range of BMI. However, because obesity or overweight is associated with increased risks for several other cancers, maintaining a normal range of BMI is currently recommended.

The major strength of our study is that it is a very large, national, population-based study. However, one limitations of this study is that the cohort data for the general population were not divided according to the histological type or stage of OC. However, since 95% of OC cases in Korea have been reported to be OSCC [2], most OC cases in this study are assumed to be OSCC. Furthermore, it was not possible to collect exact information on the TNM stage or symptoms at diagnosis. However, the present study is a cohort study that can be followed, and not a cross-sectional study. Since the median time for OC development can be as long as 4 years, it is unlikely that cases of low BMI at the time of enrolment could be attributable solely due to dysphasia or advanced disease. The median survival time for patients receiving radiation therapy or combined chemotherapy plus radiotherapy is reportedly less than 1.5 years [38]. Moreover, after excluding the first 2 years of follow-up, the finding that low BMI was associated with an increased risk of developing OC remained unchanged (S1 Table). Finally, the huge sample size in the present is associated with the possibility of overestimation of the hazard ratios. However, our previous pilot study with 264,084 individuals, who were selected as a 2% standardized sample, showed consistent results in terms of the association between low BMI and the increased OC risk (data not shown). The effect of GGT values on increased risk for OC was significant after controlling several confounding factors, and similar HR was observed in other study [11].

In conclusion, elevated GGT values were associated with an increased risk of developing OC in the general Korean population, regardless of smoking or alcohol consumption habits. Moreover, the risk was more prominent in underweight subjects. However, it should be interpreted cautiously due to a relatively modest impact compared with male sex, increased age or drinking. The role of GGT as a predictive marker for OC requires further validation.

## Supporting information

S1 Table Multivariable Analyses of the Impact of the Serum GGT Level and BMI on the Risk of Oesophageal Cancer in the General Korean Population after excluding the First 2 Years of Follow-up. (DOCX)

S2 Table Impact of the GGT Level and Underweight Status on the Risk of Oesophageal Cancer in the General Korean Population according to Different Age Ranges. (DOCX)

S3 Table Subgroup Analyses of the Impact of the GGT Level or BMI on the Risk of Oesophageal Cancer in the General Korean Population. (DOCX)
