# Supplementary material for: Elevated serum gamma-glutamyltransferase is associated with an increased risk of oesophageal carcinoma in a cohort of 8,388,256 Korean subjects
Source: PLoS One. 2017 May 5;12(5):e0177053. doi: 10.1371/journal.pone.0177053 (PMC5419599; doi:10.1371/journal.pone.0177053)
Supplement: S1 Table — (DOCX) [file pone.0177053.s001.docx]

**S1 Table. Multivariable Analyses of the Impact of the Serum GGT Level and BMI on the Risk of Oesophageal Cancer in the General Korean Population after excluding the First 2 Years of Follow-up**

|  |  | | | HR (95% CI) | |
| --- | --- | --- | --- | --- | --- |
| Variable | Event | Duration | IR^a^ | Model1^b^ | Model2^b^ |
| **GGT**^d^ |  |  |  |  |  |
| Q1 | 511 | 19696962.91 | 0.2594 | 1.00(ref.) | 1.00(ref.) |
| Q2 | 742 | 16597176.31 | 0.4471 | 1.05 (0.93-1.17) | 1.07 (0.95-1.20) |
| Q3 | 1289 | 18651843.5 | 0.6911 | 1.26 (1.13-1.4) | 1.30 (1.17-1.44) |
| Q4 | 2572 | 18002985.71 | 1.4287 | 2.43 (2.20-2.68) | 2.36 (2.13-2.62) |
| **BMI** |  |  |  |  |  |
| <18.5 | 252 | 1552344.54 | 1.6234 | 1.36 (1.19-1.55) | 1.36 (1.19-1.55) |
| 18.5-23 | 2276 | 26296746.52 | 0.8655 | 1.00(ref.) | 1.00(ref.) |
| 23-25 | 1285 | 20014746 | 0.6420 | 0.72 (0.67-0.77) | 0.72 (0.67-0.77) |
| 25-30 | 1225 | 22884976.81 | 0.5353 | 0.61 (0.57-0.65) | 0.61 (0.56-0.65) |
| 30- | 76 | 2200154.57 | 0.3454 | 0.55 (0.44-0.69) | 0.52 (0.42-0.66) |

GGT, gamma-glutamyltransferase; BMI, body mass index; HR, hazard ratio; CI, confidential interval.

^a^ Per 10,000 person-year

^b^Model 1: Adjusted for age, sex

^c^Model 2: Adjusted for age, sex, BMI, smoking, drinking, exercise, income, residence locale, diabetes, hypertension, and dyslipidaemia.

^d^GGT values were stratified into 4 quartiles: ≤16, 17–23, 24–39, and ≥40 IU/L
