# Supplementary material for: Elevated serum gamma-glutamyltransferase is associated with an increased risk of oesophageal carcinoma in a cohort of 8,388,256 Korean subjects
Source: PLoS One. 2017 May 5;12(5):e0177053. doi: 10.1371/journal.pone.0177053 (PMC5419599; doi:10.1371/journal.pone.0177053)
Supplement: S2 Table — (DOCX) [file pone.0177053.s002.docx]

**Supplementary Table** 2. Impact of the GGT level and underweight on the risk of oesophageal cancer in Korean population according to different age ranges

| Age range(years) | 40-44* | 45-49* | 50-54 | 55-59 | 60-64 | 65-69 | 70-74 | 75-79 | 80- |
| --- | --- | --- | --- | --- | --- | --- | --- | --- | --- |
| Q4(-)UW(-) | 1 | 1 | 1 | 1 | 1 | 1 | 1 | 1 | 1 |
| Q4(+)UW(-) | 1.47 | 3.667 | 3.506 | 2.511 | 2.443 | 0.875 | 1.889 | 1.242 | 1.435 |
| Q4(-)UW(+) | 2.381 | 1.751 | 1.878 | 1.965 | 1.978 | 1.572 | 1.474 | 1.555 | 1.117 |
| Q4(+)UW(+) | 12.185 | 8.354 | 7.89 | 2.714 | 6.105 | 3.414 | 2.855 | 2.278 | 0.933 |
| Q4: Serum GGT ≥40 IU/L, UW: BMI <18.5 kg/m2  * 40-49 years: HR 11.678, 95% CI 6.51-20.96 | | | | | | | | | |
