# Supplementary material for: Elevated serum gamma-glutamyltransferase is associated with an increased risk of oesophageal carcinoma in a cohort of 8,388,256 Korean subjects
Source: PLoS One. 2017 May 5;12(5):e0177053. doi: 10.1371/journal.pone.0177053 (PMC5419599; doi:10.1371/journal.pone.0177053)
Supplement: S3 Table — (DOCX) [file pone.0177053.s003.docx]

**Supplementary Table 3**. Subgroup analyses for investigating the impact of the GGT level or BMI on the risk of oesophageal cancer in Korean population

| Subgroup | GTT(quartile) | Event | HR (95% CI) |  | BMI (Kg/m^2^) | Event | HR (95% CI) |
| --- | --- | --- | --- | --- | --- | --- | --- |
| < 55years | Q1 | 124 | 1 (ref.) |  | <18.5 | 67 | 2.477(1.921,3.192) |
|  | Q2 | 150 | 0.947(0.742,1.209) |  | 18.5-23 | 570 | 1 (ref.) |
|  | Q3 | 288 | 1.161(0.925,1.457) |  | 23-25 | 332 | 0.637(0.556,0.73) |
|  | Q4 | 765 | 2.237(1.797,2.784) |  | 25-30 | 327 | 0.5(0.435,0.575) |
|  |  |  |  |  | 30- | 31 | 0.572(0.397,0.824) |
| ≥55years | Q1 | 593 | 1 (ref.) |  | <18.5 | 317 | 1.378(1.225,1.55) |
|  | Q2 | 908 | 1.1(0.991,1.221) |  | 18.5-23 | 2546 | 1 (ref.) |
|  | Q3 | 1487 | 1.271(1.151,1.402) |  | 23-25 | 1350 | 0.703(0.657,0.751) |
|  | Q4 | 2548 | 2.105(1.911,2.32) |  | 25-30 | 1250 | 0.591(0.551,0.634) |
|  |  |  |  |  | 30- | 73 | 0.522(0.413,0.66) |
| Men | Q1 | 495 | 1 (ref.) |  | <18.5 | 344 | 1.363(1.218,1.526) |
|  | Q2 | 901 | 1.122(1.005,1.252) |  | 18.5-23 | 2894 | 1 (ref.) |
|  | Q3 | 1645 | 1.341(1.211,1.485) |  | 23-25 | 1549 | 0.697(0.655,0.742) |
|  | Q4 | 3233 | 2.336(2.115,2.58) |  | 25-30 | 1410 | 0.569(0.532,0.607) |
|  |  |  |  |  | 30- | 77 | 0.475(0.379,0.597) |
| Women | Q1 | 222 | 1 (ref.) |  | <18.5 | 40 | 2.261(1.607,3.181) |
|  | Q2 | 157 | 1.031(0.838,1.267) |  | 18.5-23 | 222 | 1 (ref.) |
|  | Q3 | 130 | 1.141(0.913,1.427) |  | 23-25 | 133 | 0.838(0.675,1.04) |
|  | Q4 | 80 | 1.485(1.138,1.938) |  | 25-30 | 167 | 0.863(0.702,1.059) |
|  |  |  |  |  | 30- | 27 | 1.079(0.719,1.619) |
| Non-smoker | Q1 | 528 | 1 (ref.) |  | <18.5 | 190 | 1.527(1.312,1.777) |
|  | Q2 | 708 | 1.103(0.984,1.236) |  | 18.5-23 | 1624 | 1 (ref.) |
|  | Q3 | 1096 | 1.328(1.192,1.48) |  | 23-25 | 1068 | 0.769(0.711,0.831) |
|  | Q4 | 1738 | 2.315(2.08,2.575) |  | 25-30 | 1104 | 0.675(0.624,0.731) |
|  |  |  |  |  | 30- | 84 | 0.701(0.562,0.875) |
| Current/ex | Q1 | 189 | 1 (ref.) |  | <18.5 | 194 | 1.306(1.123,1.519) |
|  | Q2 | 350 | 1.017(0.852,1.215) |  | 18.5-23 | 1492 | 1 (ref.) |
|  | Q3 | 679 | 1.139(0.966,1.342) |  | 23-25 | 614 | 0.633(0.576,0.696) |
|  | Q4 | 1575 | 1.913(1.632,2.243) |  | 25-30 | 473 | 0.467(0.42,0.519) |
|  |  |  |  |  | 30- | 20 | 0.294(0.189,0.459) |
| Non-drinker | Q1 | 560 | 1 (ref.) |  | <18.5 | 188 | 1.484(1.272,1.732) |
|  | Q2 | 699 | 1.074(0.959,1.201) |  | 18.5-23 | 1330 | 1 (ref.) |
|  | Q3 | 915 | 1.224(1.097,1.366) |  | 23-25 | 742 | 0.745(0.681,0.816) |
|  | Q4 | 892 | 1.837(1.64,2.058) |  | 25-30 | 740 | 0.667(0.608,0.731) |
|  |  |  |  |  | 30- | 66 | 0.783(0.61,1.005) |
| Drinker | Q1 | 157 | 1 (ref.) |  | <18.5 | 196 | 1.38(1.189,1.601) |
|  | Q2 | 359 | 1.104(0.915,1.333) |  | 18.5-23 | 1786 | 1 (ref.) |
|  | Q3 | 860 | 1.31(1.103,1.556) |  | 23-25 | 940 | 0.677(0.625,0.734) |
|  | Q4 | 2421 | 2.278(1.931,2.686) |  | 25-30 | 837 | 0.528(0.485,0.575) |
|  |  |  |  |  | 30- | 38 | 0.353(0.256,0.488) |
| Non DM | Q1 | 626 | 1 (ref.) |  | <18.5 | 335 | 1.378(1.228,1.545) |
|  | Q2 | 905 | 1.094(0.986,1.213) |  | 18.5-23 | 2662 | 1 (ref.) |
|  | Q3 | 1500 | 1.314(1.192,1.449) |  | 23-25 | 1394 | 0.721(0.675,0.769) |
|  | Q4 | 2658 | 2.272(2.065,2.5) |  | 25-30 | 1229 | 0.592(0.552,0.634) |
|  |  |  |  |  | 30- | 69 | 0.522(0.41,0.664) |
| DM | Q1 | 91 | 1 (ref.) |  | <18.5 | 49 | 1.734(1.289,2.333) |
|  | Q2 | 153 | 1.076(0.829,1.397) |  | 18.5-23 | 454 | 1 (ref.) |
|  | Q3 | 275 | 1.15(0.903,1.466) |  | 23-25 | 288 | 0.636(0.548,0.738) |
|  | Q4 | 655 | 1.905(1.508,2.406) |  | 25-30 | 348 | 0.556(0.482,0.642) |
|  |  |  |  |  | 30- | 35 | 0.562(0.397,0.796) |
| Non obesity | Q1 | 605 | 1 (ref.) |  |  |  |  |
|  | Q2 | 837 | 1.038(0.934,1.154) |  |  |  |  |
|  | Q3 | 1329 | 1.223(1.107,1.352) |  |  |  |  |
|  | Q4 | 2411 | 2.195(1.993,2.419) |  |  |  |  |
|  |  |  |  |  |  |  |  |
| Obesity | Q1 | 112 | 1 (ref.) |  |  |  |  |
|  | Q2 | 221 | 1.082(0.631,1.362) |  |  |  |  |
|  | Q3 | 446 | 1.113(0.898,1.378) |  |  |  |  |
|  | Q4 | 902 | 1.687(1.366,2.084) |  |  |  |  |

Q: quartile of serum gamma glutamyltransferase; Q1: ≤16, Q2:17-23, Q3:24-39, and Q4:≥40 IU/L.

BMI, body mass index; IR, incidence rate; HR, hazard ratios; CI, confidential intervals
